# Unraveling metabolic signatures in SARS-CoV-2 variant infections using multiomics analysis

**DOI:** 10.3389/fimmu.2024.1473895

**Published:** 2024-12-11

**Authors:** Sunho Lee, Jueun Lee, Kwang-Soo Lyoo, Yourim Shin, Dong-Min Shin, Jun-Won Kim, Jeong-Sun Yang, Kyung-Chang Kim, Joo-Yeon Lee, Geum-Sook Hwang

**Affiliations:** ^1^ Integrated Metabolomics Research Group, Metropolitan Seoul Center, Korea Basic Science Institute, Seoul, Republic of Korea; ^2^ Graduate School of Pharmaceutical Sciences, College of Pharmacy, Ewha Womans University, Seoul, Republic of Korea; ^3^ College of Health Sciences, Wonkwang University, Iksan, Republic of Korea; ^4^ Bioinformatics Department, Theragen Bio, Seongnam, Republic of Korea; ^5^ National Institute of Infectious Diseases, Korea National Institute of Health, Korea Disease Control and Prevention Agency, Cheongju, Republic of Korea; ^6^ College of Pharmacy, Chung-Ang University, Seoul, Republic of Korea

**Keywords:** SARS-CoV-2, metabolomics, transcriptomics, metabolic pathway, immune response

## Abstract

**Introduction:**

The emergence of severe acute respiratory syndrome coronavirus 2 (SARS-CoV-2) variants, notably delta and omicron, has significantly accelerated the global pandemic, worsening conditions worldwide. However, there is a lack of research concerning the molecular mechanisms related to immune responses and metabolism induced by these variants.

**Methods:**

Here, metabolomics combined with transcriptomics was performed to elucidate the immunometabolic changes in the lung of hamsters infected with delta and omicron variants.

**Results:**

Both variants caused acute inflammation and lung pathology in intranasally infected hamsters. Principal component analysis uncovered the delta variant significantly altered lung metabolite levels between the pre- and post-infection states. Additionally, metabolic pathways determined by assessment of metabolites and genes in lung revealed significant alterations in arginine biosynthesis, glutathione metabolism, and tryptophan metabolism upon infection with both variants and closely linked to inflammatory cytokines, indicating immune activation and oxidative stress in response to both variants. These metabolic changes were also evident in the serum, validating the presence of systemic alterations corresponding to those identified in lung. Notably, the delta variant induced a more robust metabolic regulation than the omicron variant.

**Discussion:**

The study suggests that multi-omics is a valuable approach for understanding immunometabolic responses to infectious diseases, and providing insights for effective treatment strategies.

## Introduction

Among severe acute respiratory syndrome coronavirus-2 (SARS-CoV-2) variants, delta (B.1.617.2) and omicron (B.1.1.529) variant viruses caused a worldwide pandemic due to increased transmissibility compared to that of the Wuhan virus (1). It is urgent to elucidate the molecular mechanisms governing the onset and progression of these variants to develop effective strategies aimed at reducing recurrence rates and improving therapeutic potency.

Metabolomics has the potential to enhance our understanding of host−pathogen interactions in infectious diseases. In particular, metabolomics has been widely applied for biomarker discovery and to investigate the immunometabolic response of individuals infected with various viruses, including more recently SARS-CoV-2 (2–7). Notably, targeting specific metabolic pathways that are crucial for viral replication can potentially disrupt virus growth and reduce infection severity (8). Depletion of GSH due to viral infection leads to disruption of the redox balance in the lungs and results in tissue damage (9). High kynurenine/tryptophan ratios were observed in the plasma of patients with moderate and severe COVID-19 (10). L-arginine is metabolized to L-ornithine in the urea cycle by arginase and serves as a substrate for the production of nitric oxide (NO) by nitric oxide synthase (NOS), a signaling molecule involved in inflammatory responses (11). Interestingly, arginine administration and modulation of nitric oxide (NO) production have emerged as promising therapeutic strategies with high potency in the management of patients with severe coronavirus disease 2019 (COVID-19) (12, 13), but molecular mechanistic studies regarding arginine metabolism are still limited. Therefore, deeper metabolic pathway studies based on metabolomics are crucial to fully understand the arginine-NO metabolic pathway and its implications for therapeutic interventions in patients with severe COVID-19.

The golden Syrian hamster model is valuable for studying pulmonary pathology during COVID-19 due to the high genetic similarity of these hamsters to humans (14, 15). Recent years have seen continuous efforts to explore the pathophysiology of SARS-CoV-2 infection using various animal models such as hamsters, minks, and ferrets infected with the Wuhan virus, shedding light on changes including TCA cycle, purine metabolism, pentose phosphate pathway, kynurenine pathway and triacylglycerol accumulation (16–18). Moreover, multi-omics studies have elucidated the underlying mechanism involved in SARS-CoV-2 pathophysiology such as a shift toward enhanced glycolysis (19) and significant phospholipid metabolic alterations (20). However, metabolomic studies focusing on pulmonary pathophysiology in preclinical models of SARS-CoV-2 infection are still lacking. Current research on delta and omicron variant infections focuses on transcriptional changes in inflammatory mediators and specific genes but lacks a comprehensive view of systemic metabolic alterations in host-pathogen interactions (21).

Here, we performed molecular profiling through metabolomic and transcriptomic analysis to acquire a comprehensive understanding of the systemic effects and metabolic alterations induced by SARS-CoV-2 variants, including delta and omicron. Overall, our study provided insights into how delta and omicron viruses manipulate host’s lung metabolism. We performed metabolomic profiling and integrated transcriptomic analysis, offering valuable insights into potential therapeutic targets for the treatment of SARS-CoV-2 delta and omicron variant infections in hamsters.

## Materials and methods

### Experimental model and study design

**Figure 1:**
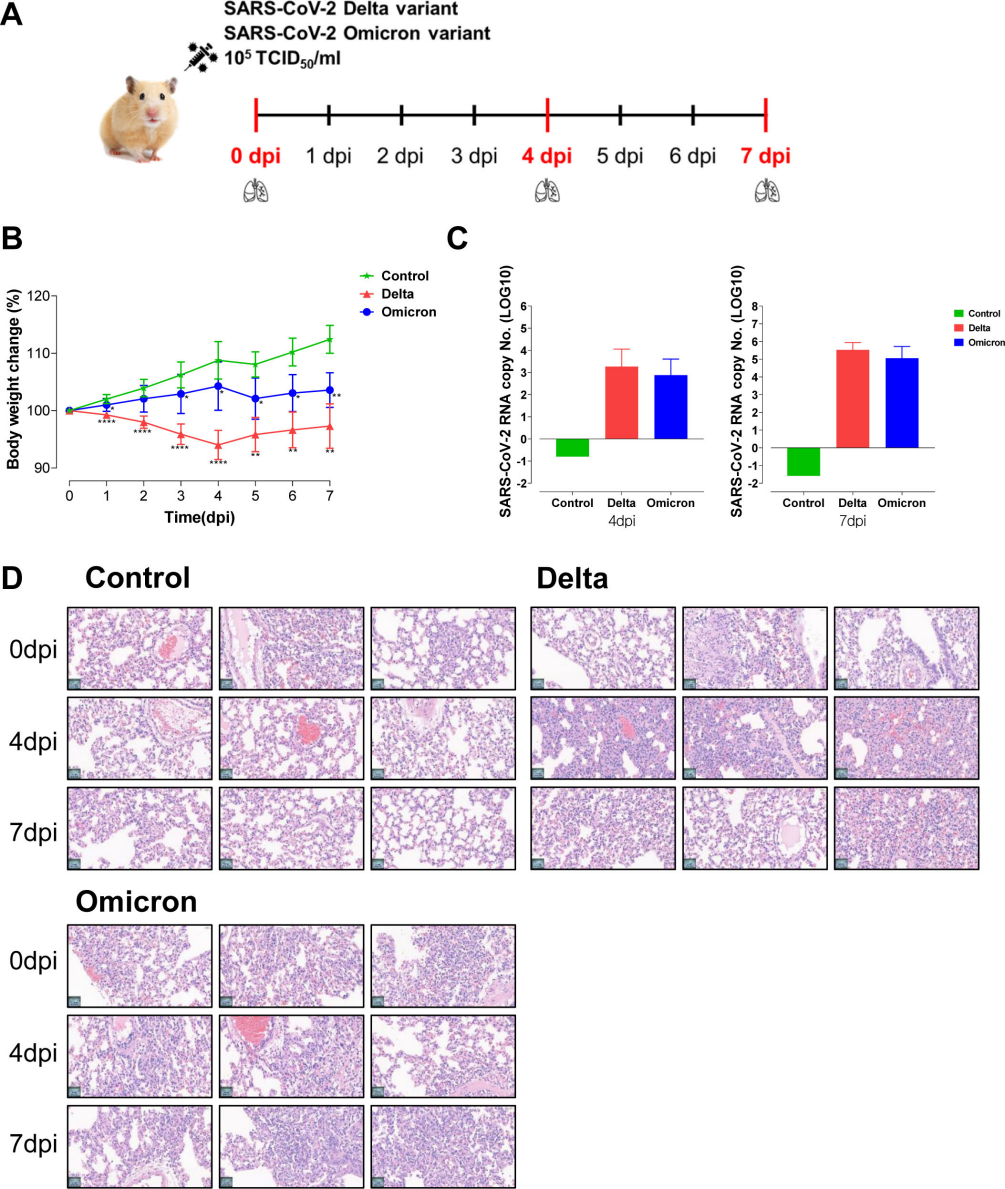
Hamsters infected with the delta and omicron variants exhibit an acute inflammatory response and lung pathology. **(A)** Study design of the Golden Syrian hamster infection model. Hamsters (n=5 per group) were intranasally infected with SARS-CoV-2 variants, specifically delta and omicron. The hamsters were sacrificed at 0 dpi, 4 dpi, and 7 dpi to obtain lung tissues for metabolic and transcriptomic analysis. **(B)** Body weight changes in hamsters after viral infection. Significant differences in delta and omicron variant groups compared to control group on each day are denoted using Mann-Whitney test by *p < 0.05, **p < 0.01, ****p < 0.0001. **(C)** The fold changes of lung viral load after delta and omicron infections compared to control group. **(D)** The histopathological lesions in the lungs assessed by using hematoxylin and eosin (H&E) staining.

Golden Syrian hamsters (6 weeks old, male) were purchased from Central Laboratory Animal Inc. (Seoul, South Korea). Our study examined male hamsters because male animals exhibited less variability in phenotype. The animals were maintained under a 12 h light and dark cycle and fed a standard diet and water ad libitum. The hamsters were divided into three groups (n=5/group): the negative control, delta variant and omicron variant groups. The hamsters were anesthetized, and thereafter, the infection was established by intranasally administration of 20 μL (10^5.0^ TCID_50_/ml) of SARS-CoV-2 delta variant (B.1.617.2) or SARS-CoV-2 omicron variant (B.1.1.529). The body weights of all infected hamsters were monitored daily until sacrifice. Five hamsters from each group were sacrificed at 0, 4, and 7 days post-infection (dpi), and the lungs were collected to assess the metabolic changes following viral infection (**Figure 1A**). Lung samples were divided for metabolic profiling, transcriptomic analysis, and H&E staining and were stored at -80°C until use. This study adhered to the guidelines of Jeonbuk National University and was approved by the Institutional Animal Care and Use Committee (approval number: JBNU 2021-086), and the experimental protocols requiring biosafety were approved by the Institutional Biosafety Committee of Jeonbuk National University (approval number: JBNU 2021-06-002-001). All animal experiments were carried out at the Animal Use Biosafety Level-3 (ABL-3) facility at the Korea Zoonosis Research Institute, which is certified by the Korea Disease Control and Prevention Agency of the Ministry of Health and Welfare (certification number: KCDC-16-3-06).

### Quantitative real-time PCR to measure the SARS-CoV-2 RNA copy number

To measure the viral loads of SARS-CoV-2 in lung tissue samples, quantitative real-time PCR was performed to detect the N gene of SARS-CoV-2 using TaqMan Fast Virus 1-Step Master Mix (Thermo Fisher Scientific, MA, USA) as previously described (22, 23). One gram of tissue samples from all hamsters were placed into soft tissue homogenizing CK14 tubes (Precellys, Betin Technologies) prefilled with ceramic beads and DMEM and then homogenized using a Bead blaster 24 (Benchmark Scientific, NJ, USA). Viral RNA was extracted from the homogenized tissues using a QIAamp viral RNA Mini Kit (Qiagen) according to the manufacturer’s protocol. Real-time PCR was conducted using a CFX96 Touch Real-Time PCR Detection System (Bio-Rad, Hercules, CA, USA).

### Histopathological analysis

All animals were euthanized using an intraperitoneal injection of xylazine and succinyl choline at the end of the experiment. At necropsy, gross lesions in the lung were examined, and then the lung tissues were collected and fixed in 4% neutral-buffered formalin for 1 week. Tissues embedded in paraffin blocks were sectioned at a thickness of 4 μm and then mounted onto glass slides. The slides were deparaffinized in xylene, rehydrated through a series of graded 100% ethanol to distilled water and then stained with hematoxylin and eosin. All tissue samples were assessed by a blinded veterinary anatomic pathologist.

### UPLC-QTOF MS-based metabolomics

To extract metabolites from lung tissue, 100 mg of lung sample was weighed and mixed with 600 μL of methanol/water (1:1, v/v) in a 1.5 mL Eppendorf (EP) tube containing zirconium oxide beads. The mixed sample was homogenized at 5,000 rpm twice using a Precellys 24 tissue grinder (Bertin Technologies, France) and centrifuged after homogenization. After adding 600 μL of chloroform, the sample was vortexed for 1 min and incubated at 4°C for 10 min. The mixture was centrifuged at 12,700 rpm for 20 min at 4°C. For the extraction of serum metabolites, 50 μL of each serum sample were mixed with 550 μL of chloroform/methanol mixture (2:1, v/v) and vortexed for 1 min. Next, 100 μL of water was mixed with the lung and serum samples, respectively and incubated at 4°C for 10 min. The mixture centrifuged at 12,700 rpm for 20 min at 4°C. Then, 150 μL of the upper aqueous supernatant from lung tissue and 50 μL supernatant from the serum were transferred into a new 1.5 mL tube and dried using a speed vac evaporator. The dried lung and serum extracts were redissolved in 200 μL of an acetonitrile/water mixture (75:25, v/v) containing internal standards (0.1 μg/ml betaine-D_11_, 10 μg/ml glutamate-^13^C_5_, 5 μg/ml leucine-^13^C_6_, 2 μg/ml phenylalanine-^13^C_6_, 10 μg/ml succinate-^13^C_4_, 10 μg/ml taurine-^13^C_2_, and 10 μg/ml uridine-^13^C_9_, ^15^N_2_).

Liquid chromatography (LC)-electrospray ionization (ESI)-mass spectrometry (MS) analyses for metabolomics of lung tissue extracts were performed on a triple TOF™ 5600 MS/MS system (AB Sciex, Canada) combined with a UPLC system (Waters, USA). LC separations were carried out on a ZIC-HILIC column (2.1 mm × 100 mm, 3.5 μm; SeQuant, Germany). The column temperature and flow rate were set to 35°C and 0.4 mL/min, respectively. The mobile phases used were 10 mM ammonium acetate and 0.1% formic acid in water/acetonitrile (10:90, v/v) (A) and water/acetonitrile (50:50, v/v) (B). The linear gradient program was as follows: 1% B from 0 to 2 min, 1–55% B from 2 to 8 min, 55–99% B from 8 to 9 min, 99% B from 9 to 11 min, 99–1% B from 11–11.1 min, and 1% B from 11.1 to 15 min. The injection volume of the sample was 2 µL for both positive and negative ionization polarity modes. Quality control (QC) samples, which were pooled identical aliquots of the samples, were analyzed regularly throughout the run to ensure data reproducibility. The spectral data were analyzed by MarkerView™ (AB Sciex, Canada), which was used to find peaks, perform peak alignment, and generate peak tables of m/z and retention times (min). The data were normalized using the total area of the spectra. To identify reliable peaks and remove instrumental bias, peaks with coefficients of variation below 20 in QC samples were selected. Metabolites were identified by comparing the experimental data against an in-house library and the online database MS-DIAL.

### mRNA sequencing and data analysis

Total RNA from lung tissues was isolated and prepared using the TRIzol cell RNA extraction protocol. The libraries were prepared for 151 bp paired-end sequencing using a TruSeq Stranded mRNA Sample Preparation Kit (Illumina, CA, USA). Namely, mRNA molecules were purified and fragmented from 1 μg of total RNA using oligo (dT) magnetic beads. The fragmented mRNAs were synthesized as single-stranded cDNAs through random hexamer priming. By applying this single-stranded cDNA as a template for second strand synthesis, double-stranded cDNA was prepared. After the sequential processes of end repair, A-tailing and adapter ligation, cDNA libraries were amplified with polymerase chain reaction (PCR). The quality of these cDNA libraries was evaluated with an Agilent 2100 Bioanalyzer (Agilent, CA, USA). The libraries were quantified with a KAPA library quantification kit (Kapa Biosystems, MA, USA) according to the manufacturer’s library quantification protocol. Following cluster amplification of denatured templates, paired-end sequencing (2×151 bp) was performed using an Illumina NovaSeq 6000 (Illumina, CA, USA).

The adapter sequences and the ends of the reads with a Phred quality score less than 20 were trimmed, and simultaneously, the reads shorter than 50 bp were removed by using cutadapt v.2.8 (24). Filtered reads were mapped to the reference genome related to the species using the aligner STAR v.2.7.1a (25) following ENCODE standard options (refer to “Alignment” of the “Help” section in the html report) with the “-quantMode TranscriptomeSAM” option for estimation of transcriptome expression level. Gene expression estimation was performed by RSEM v.1.3.1 (26) considering the direction of the reads that correspond to the library protocol using the option –strandedness. To improve the accuracy of the measurement, the “–estimate-rspd” option was applied. All other options were set to default values. To normalize the sequencing depth among samples, FPKM and TPM values were calculated. Based on the estimated read counts in the previous step, differentially expressed genes (DEGs) were identified using the R package TCC v.1.26.0 (27). The TCC package applies robust normalization strategies to compare tag count data. Normalization factors were calculated using the iterative DESeq2 (28) method. The Q-value was calculated based on the p value using the p.adjust function of the R package with default parameter settings. The DEGs were identified based on the q-value threshold less than 0.05 for correcting errors caused by multiple testing (29).

### Network analysis

We constructed a network based on correlation coefficients among the metabolites, transcriptome, and cytokines using Cytoscape v.3.10.1 (https://cytoscape.org). In the network graph, the metabolites and transcripts within the three selected metabolic pathways and significantly altered cytokines within the SARS-CoV-2 variant group are represented as nodes. The thickness of the lines connecting each node was determined by the Pearson’s correlation coefficient values.

### Statistics

SIMCA-P+ v.16.0 (Umetrics, Sweden) was used to conduct multivariate analysis. All metabolite levels were scaled to unit variance prior to principal component analysis (PCA). PCA was applied to provide an overview of metabolomic data. All the results were analyzed using the Statistical Package for Social Sciences software, v.28.0 (SPSS Inc., USA) and plotted using GraphPad Prism, v.8 (GraphPad Software, Inc., USA). Statistical significance was assessed using one-way ANOVA with Tukey’s multiple comparisons *post hoc* test. After performing robust scaling on the metabolomics and transcriptomics data using Google Colab (colab.research.google.com), Pearson’s correlation analysis was conducted on the scaled data. Pathway analysis was performed in the MetaboAnalyst computational platform (www.metaboanalyst.ca) (30).

## Results

### Acute inflammatory response and lung pathology in hamsters infected with delta and omicron

To elucidate the immune response and pathogenic molecular mechanisms of SARS-CoV-2 variants, we used the hamster model for delta and omicron variant infection (**Figure 1A**). After intranasal infection with the variants, the body weight of the hamsters was measured daily. In comparison to the non-infected control group, the groups infected with the delta and omicron variants showed significant weight loss, indicating a successful viral infection in the hamster model according to clinical signs (**Figure 1B**). Specifically, the delta variant group demonstrated a more pronounced reduction in body weight than the omicron variant group, indicating a heightened severity of viral infection within the delta group. In addition, SARS-CoV-2 viral RNA copy numbers from lung tissue in both the delta and omicron variants showed significant increases at 4 and 7 dpi compared to those of the control group (**Figure 1C**). However, no statistically significant difference was observed in the viral load between the two variants. Next, histological analysis of lung tissue was performed to evaluate pulmonary lesions (**Figure 1D**). Histopathological changes such as perivascular inflammatory cell infiltration, pneumocyte hyperplasia, alveolar hemorrhages, and septal thickening were observed in the hamsters challenged with the delta or omicron variant at 4 dpi and 7 dpi. These findings indicate that SARS-CoV-2 variant viruses infect hamster lung tissues, with delta variant causing more significant inflammatory pathology compared to omicron variant.

### Delta and omicron infection induce metabolic alterations in hamster lung tissues

**Figure 2:**
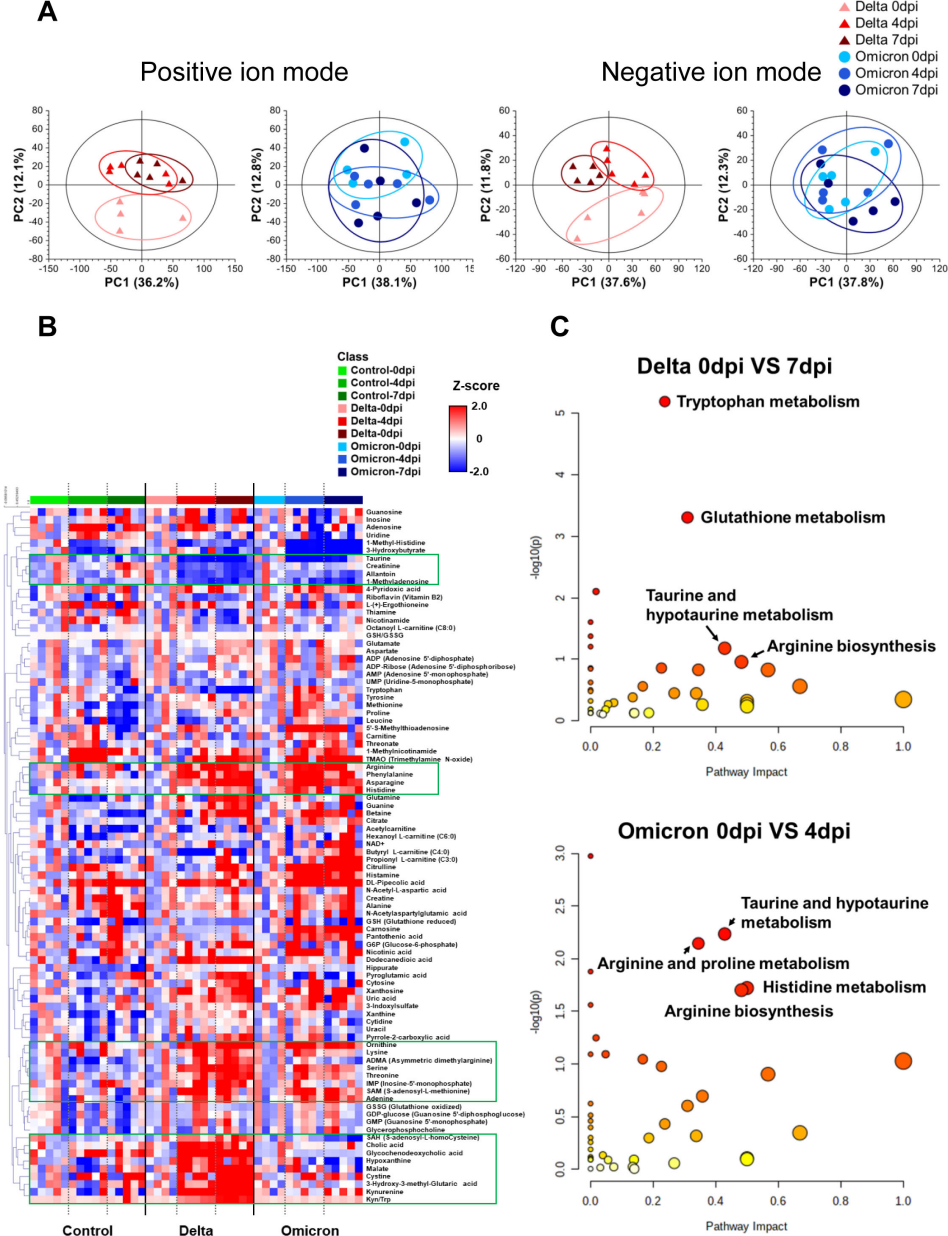
Lung metabolite profile of Golden Syrian hamsters infected with the delta and omicron variants. **(A)** Principal component analysis (PCA) score plots of lung tissue analysis shown for positive ion mode (delta variant: R^2^X = 0.482, Q^2^ = 0.257; omicron variant: R^2^X = 0.621, Q^2^ = 0.318) and for negative ion mode (delta variant: R^2^X = 0.606, Q^2^ = 0.281; omicron variant: R^2^X = 0.608, Q^2^ = 0.281). **(B)** Heat map derived for 88 identified lung metabolites showing the metabolite content changes over time after infection. **(C)** Pathway enrichment analysis of lung metabolites for infection with each variant, revealing the most associated metabolic pathways via pathway impact and adjusted p value analysis.

To investigate host-pathogen interactions and changes in host’s metabolism infected by delta and omicron variants, LC/MS-based metabolic profiling was conducted on lung tissues, a key target organ in SARS-CoV-2 pathology. A total of 5,427 and 3,110 peak features were detected in positive and negative ion modes, respectively. Tightly scattered quality control (QC) samples in principal component analysis (PCA) score plots indicated good analytical reproducibility during the LC/MS experiment (**Supplementary Figure S1A**). Regarding metabolic pattern recognition after infection with the delta variant, PCA score plots showed distinct separation between pre- and post-infection in both positive and negative ion modes (**Figure 2A**), while lung tissue samples derived from hamsters infected with omicron were slightly separated between pre- and post-infection in PCA score plots. On the other hand, no significant differences were observed PCA score plots between pre- and post-infection in control group (**Supplementary Figure S1B**). These results suggest that SARS-CoV-2 variants can modulate lung metabolism, with the delta variant exhibiting a greater impact on lung metabolism reprogramming than the omicron variant.

A heat map was generated to visualize the changes in the levels of 88 identified metabolite in the lung tissues of hamsters infected with the delta and omicron variants of SARS-CoV-2 (**Figure 2B**). In both the delta and omicron groups, we observed significant elevation of the levels of several amino acids, including arginine, phenylalanine, asparagine, histidine, tryptophan, cystine, lysine, ornithine, serine, threonine and S-adenosyl-L-methionine (SAM), after variant infection. On the other hand, there were lower levels of taurine, allantoin and 1-methyladenosine after delta and omicron infection. Interestingly, the levels of S-adenosyl-L-homocysteine (SAH), cholic acid, glycochenodeoxycholic acid, malate, 3-hydroxy-3-methylglutaric acid, and kynurenine and the ratio of kynurenine to tryptophan were markedly increased only after delta infection.

Next, to identify key metabolic pathways affected by SARS-CoV-2 variant infection at each distinct symptomatic phase (e.g., 7 dpi for delta and 4 dpi for omicron) (31), metabolic pathway analysis was performed based on differentially regulated metabolites specific to those time points (**Figure 2C**). The results of metabolic pathway analysis revealed distinct changes specific to each variant group. Arginine biosynthesis and taurine and hypotaurine metabolism were important metabolic pathways for both the delta and omicron variants. In the delta variant group, tryptophan metabolism and glutathione (GSH) metabolism were identified as key metabolic pathways. Conversely, the omicron variant group showed arginine and proline metabolism, as well as histidine metabolism, played significant roles following infection. These results demonstrated distinct metabolic changes occurring in the lung tissue of hamsters as a direct consequence of infection with the SARS-CoV-2 variants.

### Metabolic pathway regulation combined with mRNA levels in lungs infected with delta and omicron

**Figure 3:**
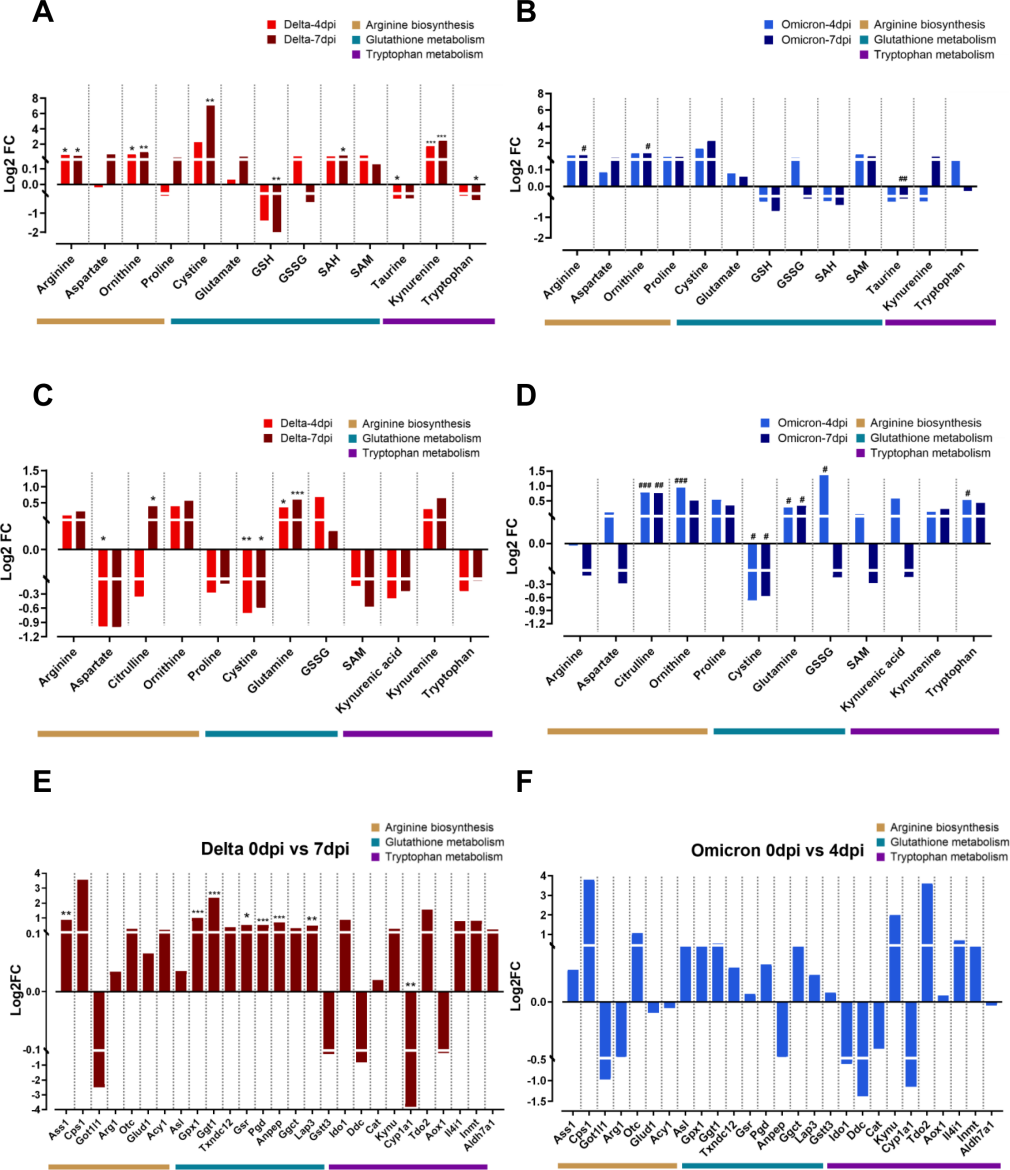
Metabolic and transcriptomic alterations in the key metabolic pathways after delta and omicron infection. The log2-fold changes in metabolite levels within significantly altered metabolic pathways after delta and omicron infections compared to those at the pre-infection state in lung tissue **(A, B)** and serum **(C, D)**. Significance of differences between pre- and post-infection in each group determined using Tukey’s multiple comparisons *post hoc* test is denoted by *p < 0.05, **p < 0.01, ***p < 0.001 for the delta group and #p < 0.05, ##p < 0.01, ###p < 0.001 for the omicron group. The log2-fold changes in gene expression within significantly altered metabolic pathways after delta **(E)** and omicron **(F)** infections compared to those at the pre-infection state. Significance of differences between pre- and post-infection in each group determined using DEG analysis is denoted by *q < 0.05, **q < 0.01, ***q < 0.001 for the delta group.

Based on the comprehensive examination of a heat map and pathway analysis, notable metabolic alterations were observed in three pathways: arginine biosynthesis, GSH metabolism, and tryptophan metabolism. The levels of most metabolites involved in arginine biosynthesis showed an increasing trend in both the delta (**Figure 3A**) and omicron (**Figure 3B**) groups compared to those pre-infection. In particular, significant accumulation of arginine and ornithine was observed after delta and omicron infection. In GSH metabolism, a remarkable increase in cystine and a decrease in GSH levels were observed in the delta variants at 7 dpi compared to those at 0 dpi. The levels of taurine were lower after delta and omicron infection than before infection. Within tryptophan metabolism, a significant increase in kynurenine levels was observed at 4 and 7 dpi, while tryptophan levels showed a decrease specifically in the delta group compared to those at baseline, indicating that tryptophan was being converted to kynurenine. To investigate systemic metabolic changes in response to coronavirus variants infections, we also examined alterations in those three pathways in the serum (**Figures 3C, D**). Increased levels of citrulline and ornithine were observed in the serum, mirroring the trends identified in lung tissue for both the delta (**Figure 3C**) and omicron groups (**Figure 3D**). Arginine levels showed an increasing trend in the serum of delta group, while a contrasting decrease was noted in omicron group. Additionally, a reduction in aspartate was observed in the serum. In the context of glutathione metabolism, a significantly reduction in cystine levels was observed in the serum, in contrast to the lung tissue. Additionally, there was an increase in both glutamine and GSSG levels in two variant groups. In tryptophan metabolism, we observed increase of kynurenine levels in both variant groups, mirroring the findings in lung tissue. The delta group exhibited a reduction in tryptophan whereas the omicron group exhibited an increase in tryptophan. Furthermore, a decline in kynurenic acid was also observed in the serum. **Supplementary Figure S2** visually represents the individual trends of metabolite levels in three specific metabolic pathways between pre- and post-infection in lung tissue and serum in both the delta and omicron groups.

We observed the correlation between the metabolic profiles of lung tissue and serum in the delta group at 7dpi (**Supplementary Figure S3A**) and the omicron group at 4dpi (**Supplementary Figure S3B**), which exhibited distinct metabolic changes after infection. In the delta group, predominantly positive correlations were observed among various metabolites (**Supplementary Figure S3A**). Notably, arginine in lung tissue were positively correlated with arginine, glutamine and kynurenine in serum. Cystine in lung tissue were positively correlated with arginine, citrulline, proline, cystine and SAM in serum. Lung kynurenine also showed positive correlation with serum citrulline, proline and cystine. Conversely, in the omicron group, predominantly negative correlations were observed among various metabolites (**Supplementary Figure S3B**). Particularly, proline in lung tissue showed a significant negative correlation with ornithine, GSSG and SAM in serum. These findings underscore that the delta and omicron variants induce different metabolic alterations in the host’s lung tissue and serum following infection, and imply that a coronavirus infection impacts not only the pulmonary tissue but also has systemic effects throughout the body.

Next, an RNA-Seq analysis was conducted to investigate the transcriptional alterations in genes linked to each of the three identified metabolic pathways, as outlined in the Kyoto Encyclopedia of Genes and Genomes (KEGG). The genes associated with the three metabolic pathways showed mostly similar trends of changes in transcription for both the delta and omicron variants, especially the magnitude of the significant change, which was much larger in the delta group than in the omicron group (**Figures 3E, F**). In arginine biosynthesis, the levels of Ass1 were significantly increased at 7 dpi compared to those at 0 dpi in the delta group but not in the omicron group. The transcription level of most genes involved in GSH metabolism, including Gpx1, Ggt1, Gsr, Pgd, Anpep and Lap3, was significantly higher post-infection than pre-infection in the delta group but not in the omicron group. In tryptophan metabolism, increased patterns of transcription of genes related to kynurenine synthesis, including Tdo2 and Ido1, were observed, while the levels of Cyp1a1 were significantly lower at 7 dpi than at 0 dpi in the delta group.

**Figure 4:**
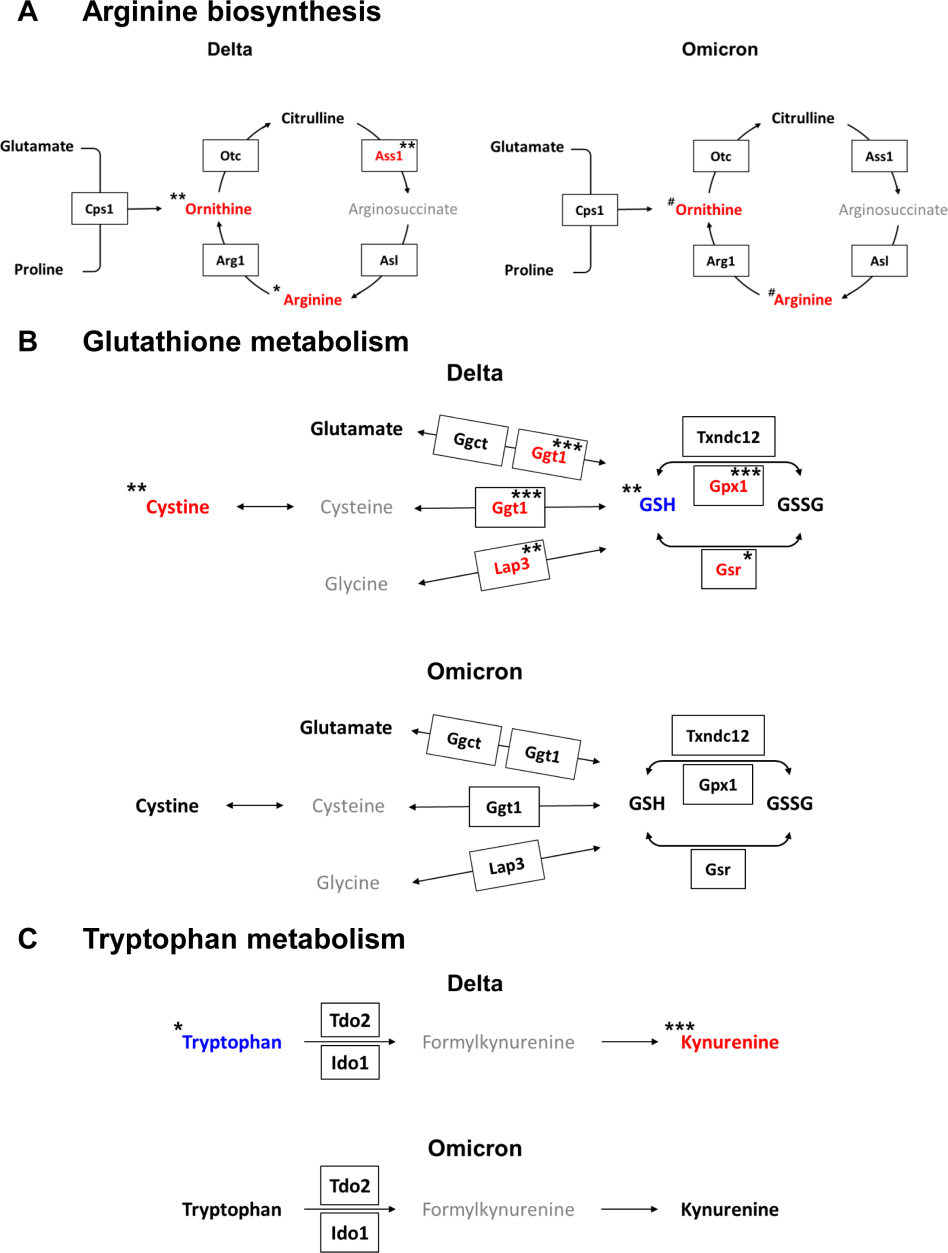
Integrated metabolic pathways combined with metabolites and genes from lungs infected with the delta and omicron variants. **(A)** Arginine biosynthesis. **(B)** Glutathione metabolism. **(C)** Tryptophan metabolism. Colored red or blue letters represent increased or decreased levels after infection for the delta variant at 7 dpi or the omicron variant at 4 dpi compared to those at 0 dpi, respectively. The pathways were modified from those in the KEGG database (http://www.genome.jp/kegg/). Significance of differences between pre- and post-infection in each group determined by using Tukey’s multiple comparisons *post hoc* test for metabolite levels (*p < 0.05, **p < 0.01, ***p < 0.001 for the delta group; #p < 0.05 for the omicron group) and for DEGs (*q < 0.05, **q < 0.01, ***q < 0.001 for the delta group).

Based on metabolomic and transcriptomic analyses, we were able to identify altered metabolic pathways in response to the SARS-CoV-2 variants. By combining the two sets of analyses, the modified metabolic pathways could be depicted in a single figure (**Figure 4**). Upregulation of arginine biosynthesis and the urea cycle was observed with both the delta and omicron variants (**Figure 4A**). An examination of the integrated metabolic pathway for GSH metabolism revealed distinct alterations in the context of the delta variant, wherein the synthesis of GSH was found to be suppressed, concomitant with an augmented production of cystine (**Figure 4B**). In the context of the metabolic pathway related to tryptophan metabolism, enhancement of the synthesis of kynurenine was observed with only the delta variant (**Figure 4C**). These findings demonstrate that SARS-CoV-2 variants induce alterations in the metabolic pathways of hamster lung tissue. Specifically, it was shown that the delta variant of the virus had a stronger impact on the lung metabolism of hamsters upon infection than the omicron variant.

### Impaired metabolic pathways were associated with inflammatory cytokines after delta and omicron infection

The levels of cytokines, including IL-6, IL-1β, IL-10, IFN-γ, tumor necrosis factor-α (TNF-α), and colony-stimulating factor (CSF), gradually increase with the severity of COVID-19 and play a crucial role in the immune response to SARS-CoV-2 infection (10, 32). Thus, the mRNA levels of cytokines were examined to gain insights into their role in the immune response to SARS-CoV-2 delta and omicron variants (**Supplementary Figure S4**). Most cytokine levels within the lung tissue were elevated after infection with the delta and the omicron variants, consistent with previous studies. In particular, we observed increase in the levels of cytokines known to contribute to cytokine storms as IL-1β, IL-6, IL-12A, IL-12B, IFN-γ, and TNF-α as well as various chemokines and CSFs upon infection with the COVID-19 variants (**Supplementary Figure S4**). Moreover, these alterations were more notable in the delta group than in the omicron group.

**Figure 5:**
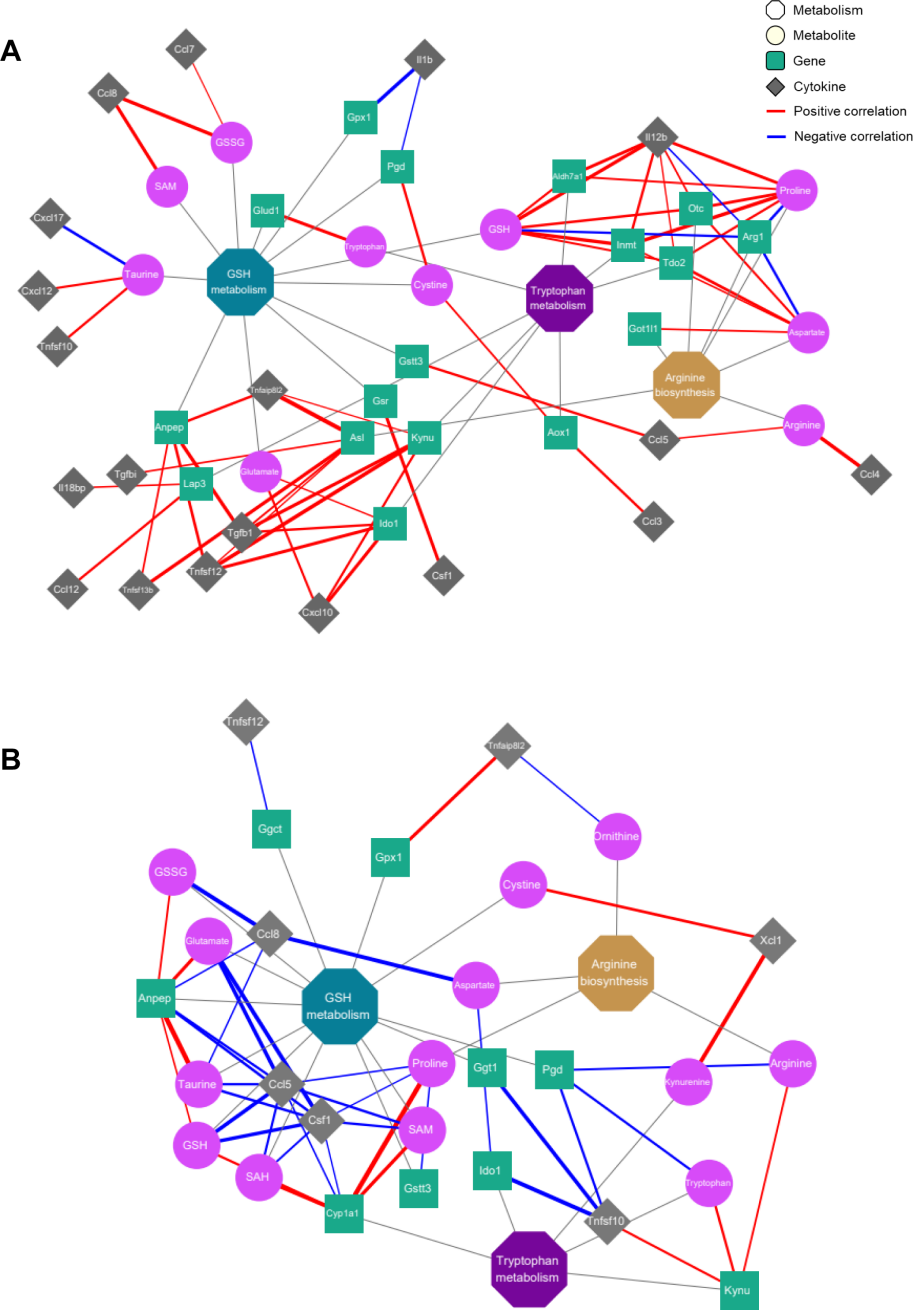
Correlation networks of inflammatory cytokines with metabolites and genes in the key metabolic pathways. Co-expression network analyses of the key regulatory metabolites and genes assigned to arginine biosynthesis, glutathione metabolism and tryptophan metabolism for the delta **(A)** and omicron **(B)** variants. Octagons, circles, squares and rhombi indicate metabolic pathways, metabolites, genes and cytokines, respectively. Edges depict the association between each node, with red or blue denoting positive or negative correlations, respectively. The thickness of each edge represents the strength of the correlation between factors.

Next, a correlation analysis was conducted to explore the association between metabolites and genes involved in infection-induced altered metabolic pathways and all cytokines changed after infection (**Supplementary Figure S5**). To visualize and interpret the modulation of metabolic pathways in relation to metabolic and transcriptional changes in the immune response, we created integrated metabolic network diagrams based on the correlation analysis of cytokines, metabolites, and genes for both the delta and omicron variants (**Figure 5**). For the delta variant, the network showed predominantly strong positive correlations among cytokines, metabolites, and transcripts (**Figure 5A**). In particular, arginine exhibited positive correlations with major proinflammatory cytokines, such as CCL4 and CCL5. And proline and GSH were positively correlated with IL-12B (**Figure 5A**; **Supplementary Figure S5A**). Additionally, oxidized glutathione (GSSG) and SAM were positively correlated with CCL8, while taurine exhibited a negative correlation with CXCL17 (**Figure 5A**; **Supplementary Figure S5A**). Regarding transcriptome profiles, strong positive correlations were observed between genes and cytokines, mirroring the correlations between metabolites and cytokines (**Figure 5A**; **Supplementary Figure S5C**). Genes related to arginine biosynthesis, such as Got1l1, Otc, and Asl, exhibited positive correlations with most cytokines. Notably, Asl showed a significant positive correlation with cytokines from the TNF and transforming growth factor-beta (TGF-β) families, while Arg1 and Otc showed negative and positive correlations with IL-12B, respectively. In GSH metabolism, Gpx1 and Pgd exhibited a negative correlation with IL-1β, while Anpep showed positive correlations with TNFAIP8L2, TNFSF12, TNFSF13b, and TGF-β1. Additionally, Lap3 was positively correlated with CCL12 and IL-18bp, and Gstt3 exhibited a positive correlation with CCL5. In tryptophan metabolism, Ido1 and Kynu showed positive correlations with CXCL10, TNFSF12, and TGF-β1, while Tdo2, Inmt, and Aldh7a1 exhibited positive correlations with IL-12B. For the omicron variant, the network primarily showed negative correlations (**Figure 5B**). Specifically, all metabolites exhibited negative correlations with CCL5, CCL8, TNFAIP8L2, and CSF1, but showed positive correlations with XCL1 (**Figure 5B**; **Supplementary Figure S5B**). No significant correlations were observed between genes related to arginine biosynthesis and cytokines for the omicron variant (**Figure 5B**; **Supplementary Figure S5D**).

In glutathione metabolism, Ggt1 and Pgd were negatively correlated with TNFSF10, while Gpx1 exhibited a positive correlation with TNFAIP8L2. Additionally, Anpep showed negative correlations with CCL5, CCL8, and CSF1. In tryptophan metabolism, Ido1 and Kynu showed negative and positive correlations with TNFSF10, respectively, while Cyp1a1 exhibited negative correlations with CCL5 and CSF1.These results indicate that SARS-CoV-2 variant infection triggers an inflammatory response associated with arginine biosynthesis, glutathione metabolism and tryptophan metabolism in the lungs of hamsters by modulating metabolite and transcript levels, and the delta and omicron variant viruses exert distinct inflammatory responses on hamster lung tissue, as evidenced by different correlations with cytokines.

## Discussion

In this study, we investigated a comprehensive molecular mechanism in hamster lung tissue infected with delta and omicron SARS-CoV-2 variants by integrating metabolomics and transcriptomics. Following viral infection, arginine biosynthesis, GSH metabolism, and tryptophan metabolism were concurrently regulated at both the metabolic and genetic levels in lung tissue. Importantly, these metabolic pathways were notably associated with the production of inflammatory cytokines. Interestingly, the delta variant induced a stronger impact on lung metabolism and inflammatory responses compared to the omicron variant, according to metabolic profile patterns (**Figure 2A**), levels of metabolites (**Figures 3A, B**) and genes (**Figures 3E, F**), and changes in cytokine levels (**Supplementary Table S3**). Additionally, these metabolic alterations were reflected in the serum, emphasizing the systemic impact of the virus on various metabolic processes. Viruses can influence host metabolic processes and induce physiological dysfunction (33). Understanding the pathophysiology of SASR-CoV-2 through the elucidation of molecular mechanisms via metabolomics and transcriptomics, as well as exploring metabolic interventions as novel therapeutic strategies, may contribute to the prevention and treatment of COVID-19. Hence, this study can provide potential molecular targets for therapeutic exploration in the quest for new drugs targeting the host pulmonary immune response following infection with delta and the omicron variants.

In this study, an increase in arginine synthesis was observed in both the delta and omicron variant viruses. Arginine serves as a substrate for the generation of nitric oxide (NO), which is a signaling molecule in inflammatory responses. Previous studies reported decreased levels of arginine and a dysregulated urea cycle in plasma from patients with severe COVID-19 (34, 35). Within the urea cycle, arginine is converted to ornithine and then recycled back to arginine via the enzymes Otc, Ass1, and Asl (36). Therefore, the increase in arginine levels can be derived from ornithine, as indicated by the upregulation of enzymes such as Arg1, Ass1, Otc, and Asl within the urea cycle. The alterations in arginine biosynthesis could be attributed to the actions of the urea cycle toward enhancing the reduction of elevated NO levels induced by the inflammation triggered by infection. Interestingly, some studies have suggested that arginine supplementation therapy in COVID-19 patients could improve immune function and reduce inflammation (34, 37–39). Additionally, targeting arginine depletion by regulating arginine biosynthesis enzymes, aiming to inhibit viral replication may present a potential therapeutic strategy for the treatment of COVID-19 patients (36).

Previously, a decrease in the levels of GSH along with an increase in the levels of GSSG was observed after coronavirus infection (40) indicating enhanced intracellular free radical generation and increased oxidative stress. Lung tissue functions as a reservoir for cellular thiols, primarily in the form of GSH. Viral infections deplete GSH and disrupt the redox balance in lung tissue, inducing cellular stress with lung damage (9). In patients experiencing hypoxemia due to SARS-CoV-2 infection, a reduction in serum cysteine has been reported, consistent with our research findings (41). Furthermore, we observed a significant increase in the levels of cystine, Ggt1 and Lap3. When GSH levels within the lung tissue are maintained, cystine from outside the cells enters and undergoes reduction to cysteine inside the cells (41, 42). The decrease in GSH levels due to viral infection is anticipated to result from a reduction in serum cysteine levels required for GSH synthesis and the inhibition of the conversion of cystine to cysteine in lung tissue, leading to the accumulation of cystine. Thus, this study suggested that the alteration in GSH metabolism during SARS-CoV-2 variant infection can serve as an indicator of how the coronavirus affects oxidative stress and contributes to lung damage.

In tryptophan metabolism, kynurenine, primarily known as an inflammatory marker, was significant enriched, along with a notable decrease in tryptophan levels after delta variant infection. Additionally, increased expression of genes such as tryptophan 2,3-dioxygenase 2 (Tdo2) and indoleamine 2,3-dioxygenase 1 (Ido1) was observed, indicating the enhancement of kynurenine synthesis after delta infection. Previous studies reported that kynurenine and tryptophan are associated with COVID-19 severity (35, 43, 44). Furthermore, Kynu and Ido1, which are involved in tryptophan metabolism, are upregulated during coronavirus infection. In particular, the reduction in tryptophan levels due to the action of Ido1 has long-term immunosuppressive effects (45). Consequently, our findings suggest that the enhancement of kynurenine synthesis represents a distinct inflammatory response in the lung tissue following infection with the delta variant.

Interestingly, our findings are consistent with those of a previous human study. Li et al. found significant up-regulation in arginine metabolism and the urea cycle as well as tryptophan metabolism in plasma samples obtained from omicron patients compared to healthy controls (46). Notably, disruption of the urea cycle was observed, with a significant increase in ornithine cycle-related metabolites such as N2-acetyl-L-ornithine and asparagine, which were associated with cytokine storm. Additionally, these findings suggested that homoarginine and ornithine play a role in liver detoxification (35). Therefore, we suggested the potential for clinical application of SARS-CoV-2 research using the hamster model.

The networks of cytokines and metabolic pathways suggested the presence of an inflammatory response and immune activation due to delta and omicron infection. Numerous studies have reported increased levels of inflammatory cytokines in COVID-19 patients, which supports our findings (32). Coronaviruses infect the respiratory tract and trigger a cytokine storm characterized by the production of inflammatory cytokines such as IL-1, IL-6, IL-8, IL-12, TNF-α, and other chemokines. This excessive release of inflammatory cytokines causes a rapid increase in cytokine levels in the bloodstream, leading to systemic inflammation. As a result, it can cause not only lung damage but also multiorgan failure, which is closely related to the severity of the disease. In patients with severe COVID-19, a high correlation was observed between circulating inflammatory cytokines, such as IL-6, CXCL10 (IP-10), and CSF1 (M-CSF), and arginine metabolism as well as tryptophan metabolism (43). Arginine is closely associated with inflammatory responses due to its essential role in T-cell activation, regulating both innate and adaptive immunity (47). These results reveal a strong correlation between TNF family cytokines and transcripts related to GSH metabolism, suggesting a potential link between the release of inflammatory cytokines and oxidative stress. The release of these inflammatory cytokines can potentially induce damage to lung tissue (48). Tryptophan metabolism is known to have the strongest correlation with IL-6 (43, 49). Additionally, TNF-α, IL-6, and IL-1β induce elevated Ido1 expression in the context of immunosuppression in lung cancer progression (50).

In conclusion, this study can be considered a notable advance as it included a comprehensive approach involving metabolic and transcriptomic profiling in animal models, which is relatively unexplored in the context of SARS-CoV-2 and its variants. We suggest that arginine biosynthesis, GSH metabolism and tryptophan metabolism are key metabolic pathways, shedding light on their relationship with the pulmonary immune response to both the delta and omicron infections. Furthermore, these pathways could be potential targets for therapeutic interventions aimed at mitigating the impact of these two SARS-CoV-2 variants. Overall, this study demonstrates that metabolic profiling with transcriptomic profiling is a valuable tool for exploring the immunometabolic responses associated with infectious diseases.

## Data Availability

The datasets presented in this study can be found in online repositories. The names of the repository/repositories and accession number(s) can be found below: KAP240957 in K-BDS (Korea BioData Station).

## Supplementary material

Supplementary Figure 1 Principal component analysis (PCA) score plots of lung tissue with quality control (QC) samples including the control, delta and omicron groups (A) for positive ion mode (R^2^X = 0.648, Q^2^ = 0.426) and negative ion mode (R^2^X = 0.706, Q^2^ = 0.502) and the control group (B) for positive ion mode (R^2^X = 0.461, Q^2^ = 0.241) and negative ion mode (R^2^X = 0.517, Q^2^ = 0.324)..

Supplementary Figure 2 Line graphs for metabolites in lung tissue and serum with significantly changed levels in three key metabolic pathways after delta and omicron infection. **(A)** Arginine biosynthesis. **(B)** Glutathione metabolism. **(C)** Tryptophan metabolism. Significance of differences between pre- and post-infection in each group determined using Tukey's multiple comparisons post hoc test is denoted by *p < 0.05, **p < 0.01, ***p < 0.001 for the delta group and ^#^p < 0.05, ^##^p < 0.01 for the omicron group.

Supplementary Figure 3 Correlation analysis of lung metabolites with serum metabolites in the delta at 7dpi **(A)** and omicron at 4dpi **(B)** infection groups. Pearson’s correlation coefficients ranged from -1.0 to +1.0 and are indicated in red and blue for positive and negative correlations, respectively. Significance of correlations is denoted by *p < 0.05, ***p < 0.005.

Supplementary Figure 4 Heat maps illustrating the log2-fold change (FC) derived for cytokine levels at 4 and 7 dpi in comparison to those at baseline at 0 dpi. CSF, colony-stimulating factor, TGF-β, transforming growth factor-beta, TNF, tumor necrosis factor.

Supplementary Figure 5 Correlation analysis of cytokines with metabolites in the delta **(A)** and omicron **(B)** infection groups, and with genes in the delta **(C)** and omicron **(D)** infection groups was performed to examine the overall relationship between cytokines and these molecular changes. Pearson’s correlation coefficients ranged from -1.0 to +1.0 and are indicated in red and blue for positive and negative correlations, respectively. Significance of correlations is denoted by *p < 0.05, **p < 0.01, ***p < 0.001.
