# Supplementary figures and images for: Unraveling metabolic signatures in SARS-CoV-2 variant infections using multiomics analysis
Source: Front Immunol. 2024 Dec 11;15:1473895. doi: 10.3389/fimmu.2024.1473895 (PMC11697598; doi:10.3389/fimmu.2024.1473895)

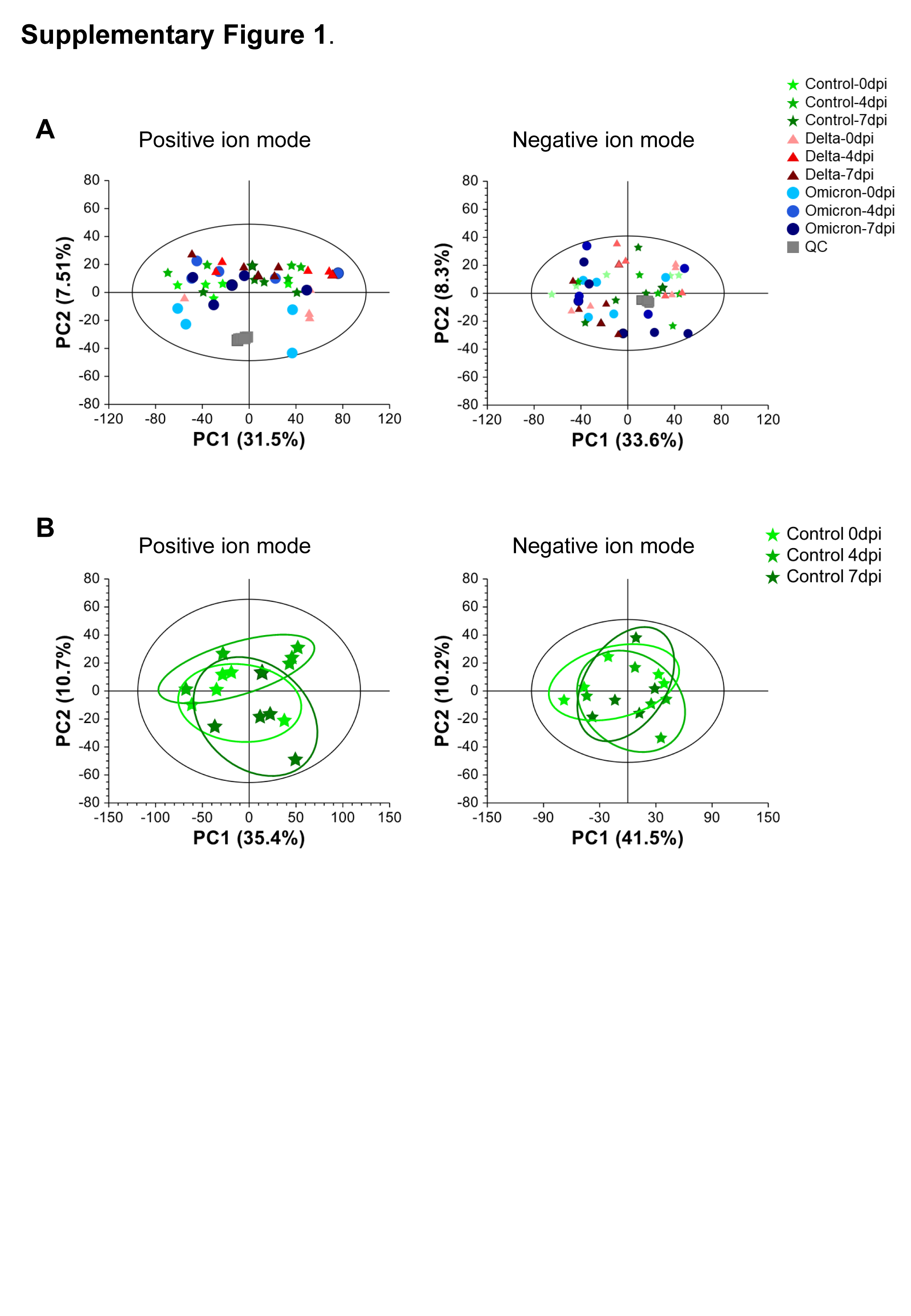

Supplement: Supplementary Figure 1 — Principal component analysis (PCA) score plots of lung tissue with quality control (QC) samples including the control, delta and omicron groups (A) for positive ion mode (R2X = 0.648, Q2 = 0.426) and negative ion mode (R2X = 0.706, Q2 = 0.502) and the control group (B) for positive ion mode (R2X = 0.461, Q2 = 0.241) and negative ion mode (R2X = 0.517, Q2 = 0.324).. [file Image1.tif]

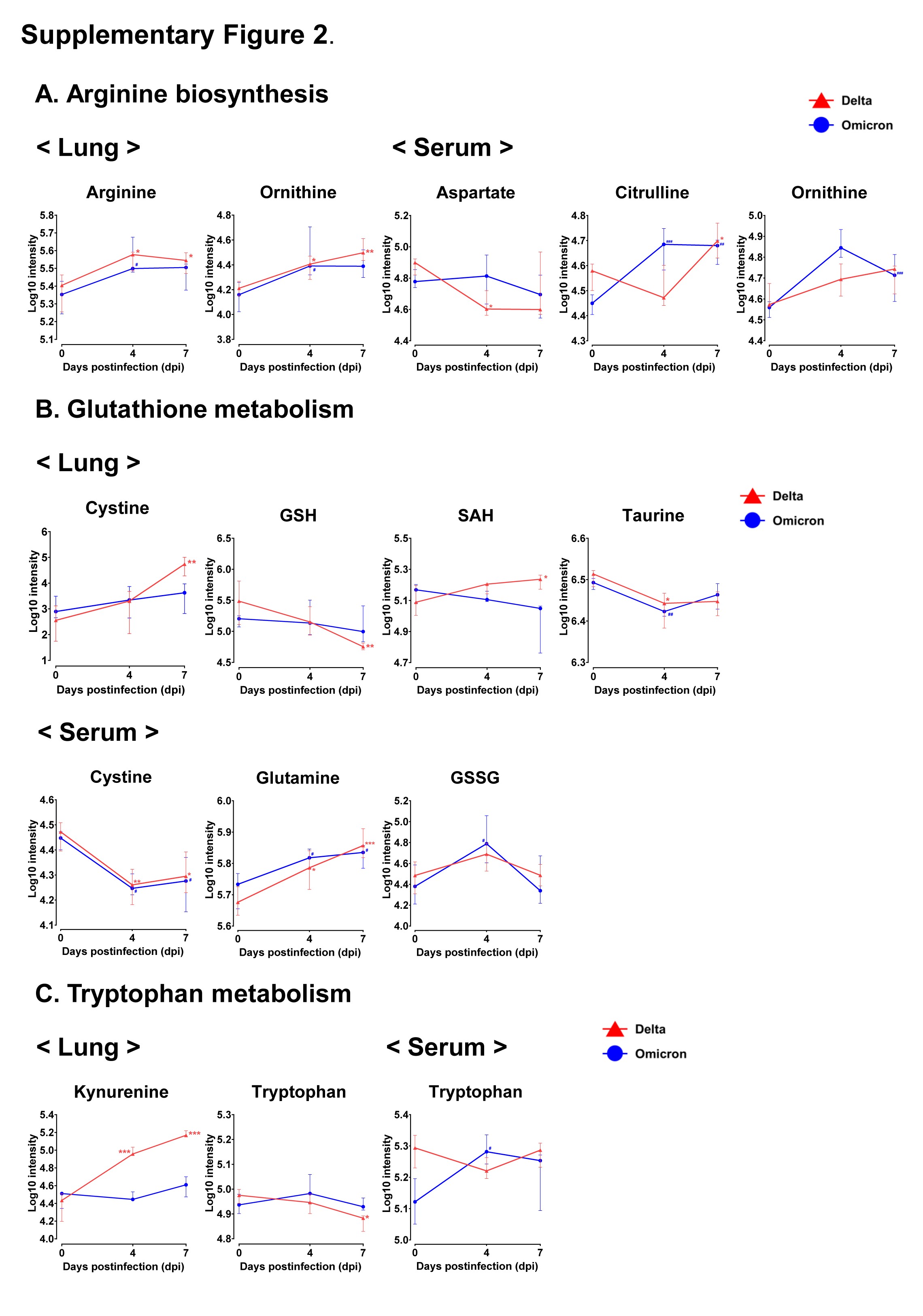

Supplement: Supplementary Figure 2 — Line graphs for metabolites in lung tissue and serum with significantly changed levels in three key metabolic pathways after delta and omicron infection. (A) Arginine biosynthesis. (B) Glutathione metabolism. (C) Tryptophan metabolism. Significance of differences between pre- and post-infection in each group determined using Tukey's multiple comparisons post hoc test is denoted by *p < 0.05, **p < 0.01, ***p < 0.001 for the delta group and #p < 0.05, ##p < 0.01 for the omicron group. [file Image2.tif]

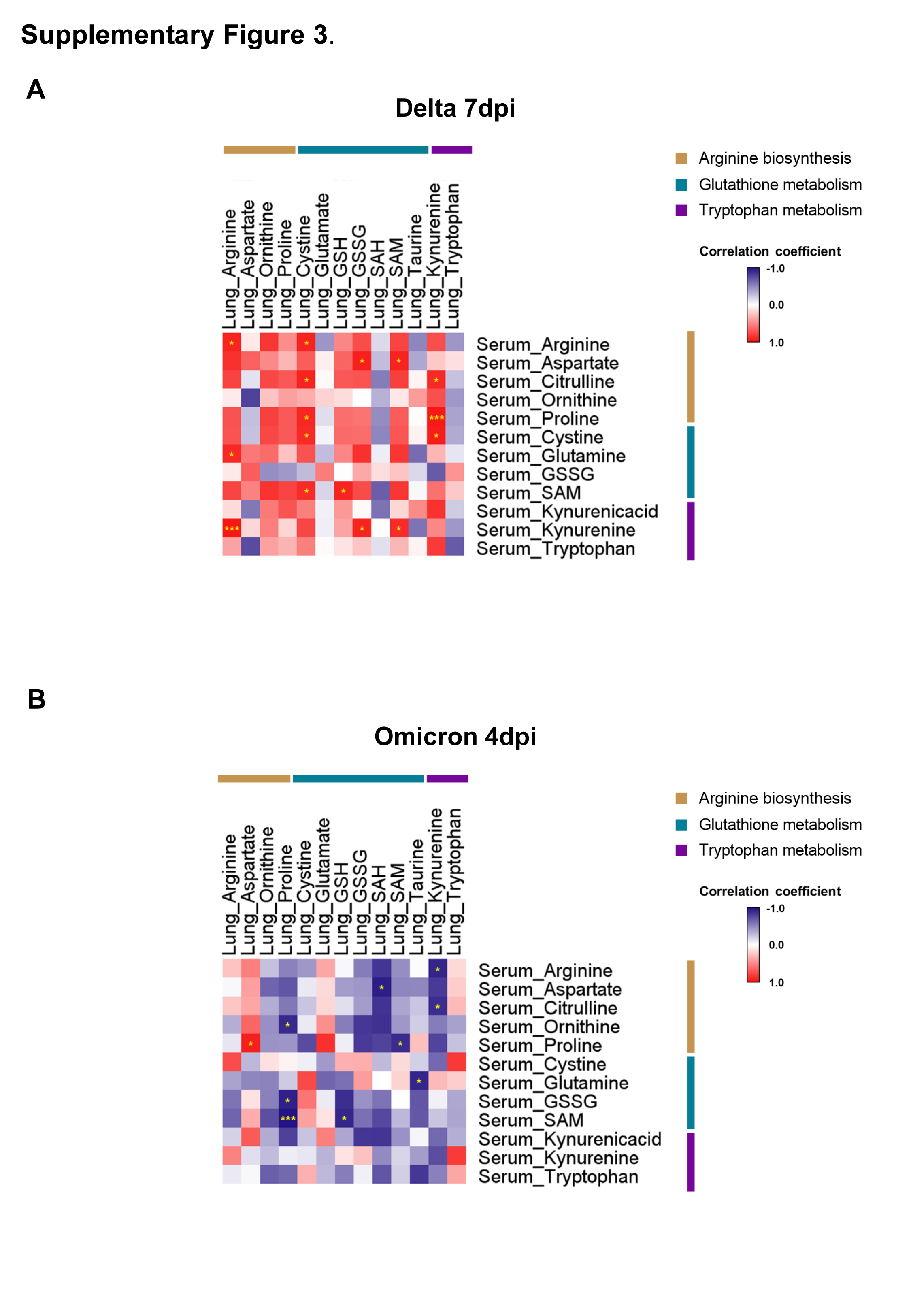

Supplement: Supplementary Figure 3 — Correlation analysis of lung metabolites with serum metabolites in the delta at 7dpi (A) and omicron at 4dpi (B) infection groups. Pearson’s correlation coefficients ranged from -1.0 to +1.0 and are indicated in red and blue for positive and negative correlations, respectively. Significance of correlations is denoted by *p < 0.05, ***p < 0.005. [file Image3.tif]

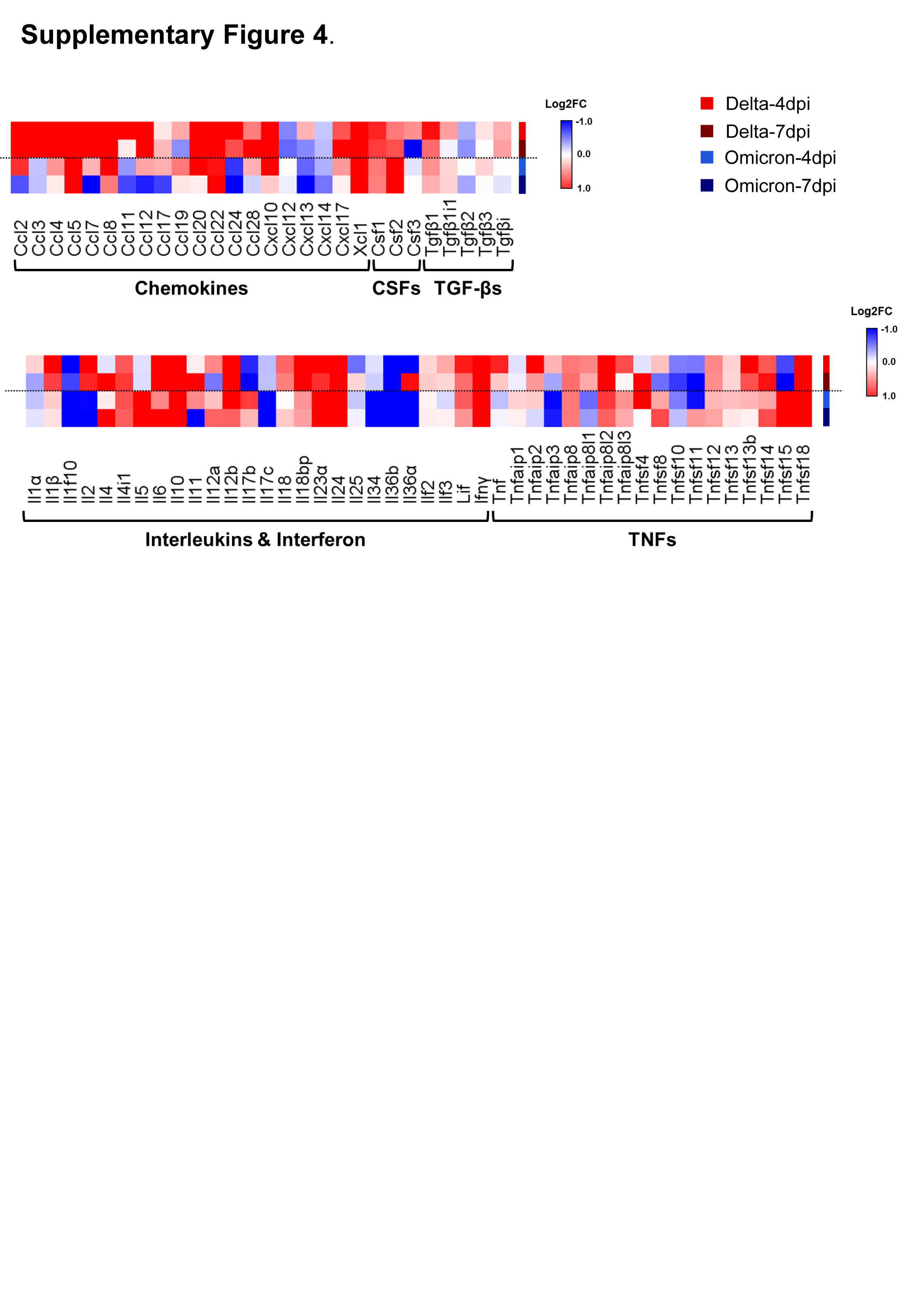

Supplement: Supplementary Figure 4 — Heat maps illustrating the log2-fold change (FC) derived for cytokine levels at 4 and 7 dpi in comparison to those at baseline at 0 dpi. CSF, colony-stimulating factor, TGF-β, transforming growth factor-beta, TNF, tumor necrosis factor. [file Image4.tif]

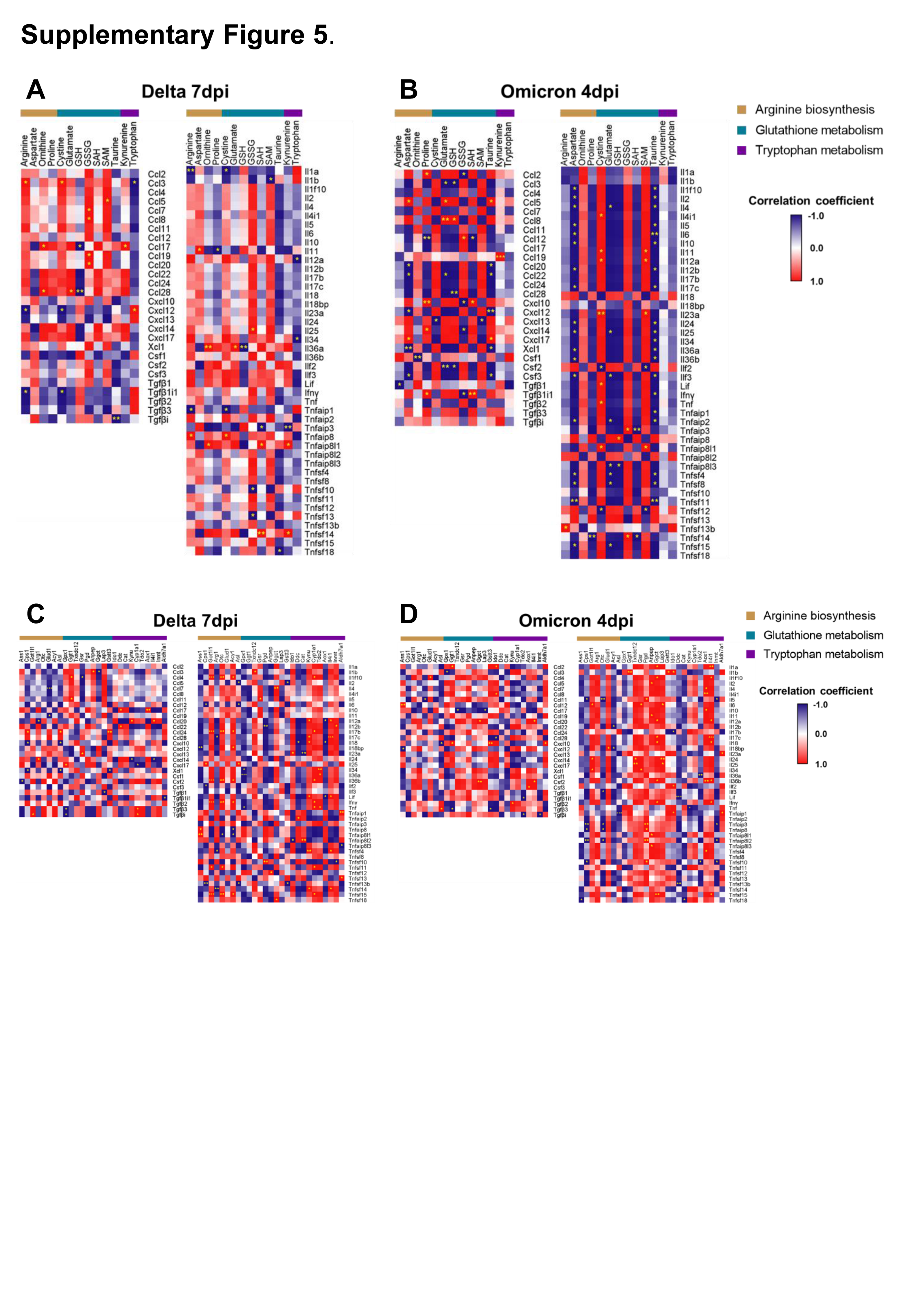

Supplement: Supplementary Figure 5 — Correlation analysis of cytokines with metabolites in the delta (A) and omicron (B) infection groups, and with genes in the delta (C) and omicron (D) infection groups was performed to examine the overall relationship between cytokines and these molecular changes. Pearson’s correlation coefficients ranged from -1.0 to +1.0 and are indicated in red and blue for positive and negative correlations, respectively. Significance of correlations is denoted by *p < 0.05, **p < 0.01, ***p < 0.001. [file Image5.tif]
